# Short-term test-retest-reliability of conditioned pain modulation using the cold-heat-pain method in healthy subjects and its correlation to parameters of standardized quantitative sensory testing

**DOI:** 10.1186/s12883-016-0650-z

**Published:** 2016-08-05

**Authors:** Julia Gehling, Tina Mainka, Jan Vollert, Esther M. Pogatzki-Zahn, Christoph Maier, Elena K. Enax-Krumova

**Affiliations:** 1 Department of Pain Medicine, Berufsgenossenschaftliches Universitätsklinikum Bergmannsheil GmbH, Ruhr-University Bochum, Bürkle-de-la-Camp-Platz 1, 44789 Bochum, Germany; 2 Department of Neurology, University Medical Center Hamburg-Eppendorf, Martinistraße 52, 20246 Hamburg, Germany; 3 Department of Anaesthesiology, Intensive Care and Pain Medicine, University Hospital Münster, Albert-Schweitzer-Campus 1, GB A1, 48149 Münster, Germany; 4 Department of Neurology, Berufsgenossenschaftliches Universitätsklinikum Bergmannsheil GmbH, Ruhr-University Bochum, Bürkle-de-la-Camp-Platz 1, 44789 Bochum, Germany

**Keywords:** Conditioned pain modulation, Test-retest reliability, Quantitative sensory test, Heat-cold-pain method, Early CPM effect, Late CPM effect

## Abstract

**Background:**

Conditioned Pain Modulation (CPM) is often used to assess human descending pain inhibition. Nine different studies on the test-retest-reliability of different CPM paradigms have been published, but none of them has investigated the commonly used heat-cold-pain method. The results vary widely and therefore, reliability measures cannot be extrapolated from one CPM paradigm to another. Aim of the present study was to analyse the test-retest-reliability of the common heat-cold-pain method and its correlation to pain thresholds.

**Methods:**

We tested the short-term test-retest-reliability within 40 ± 19.9 h using a cold-water immersion (10 °C, left hand) as conditioning stimulus (CS) and heat pain (43-49 °C, pain intensity 60 ± 5 on the 101-point numeric rating scale, right forearm) as test stimulus (TS) in 25 healthy right-handed subjects (12females, 31.6 ± 14.1 years). The TS was applied 30s before (TS_before_), during (TS_during_) and after (TS_after_) the 60s CS. The difference between the pain ratings for TS_before_ and TS_during_ represents the early CPM-effect, between TS_before_ and TS_after_ the late CPM-effect. Quantitative sensory testing (QST, DFNS protocol) was performed on both sessions before the CPM assessment. Statistics: paired t-tests, Intraclass correlation coefficient (ICC), standard error of measurement (SEM), smallest real difference (SRD), Pearson’s correlation, Bland-Altman analysis, significance level *p* < 0.05 with Bonferroni correction for multiple comparisons, when necessary.

**Results:**

Pain ratings during CPM correlated significantly (ICC: 0.411…0.962) between both days, though ratings for TS_after_ were lower on day 2 (*p* < 0.005). The early (day 1: 16.7 ± 11.7; day 2: 19.5 ± 11.9; ICC: 0.618, SRD: 20.2) and late (day 1: 1.7 ± 9.2; day 2: 7.6 ± 11.5; ICC: 0.178, SRD: 27.0) CPM effect did not differ significantly between both days. Both early and late CPM-effects did not correlate with the pain thresholds.

**Conclusions:**

The short-term test-retest-reliability of the early CPM-effect using the heat-cold-pain method in healthy subjects achieved satisfying results in terms of the ICC. The SRD of the early CPM effect showed that an individual change of > 20 NRS can be attributed to a real change rather than chance. The late CPM-effect was weaker and not reliable.

## Background

Human pain modulation is of growing interest for pain research. The balance between inhibitory and facilitatory pain systems is suggested to be disrupted in several pain syndromes, e.g. fibromyalgia, irritable bowel syndrome or osteoarthritis [1–3]. Furthermore, ineffective endogenous analgesia seems to put patients at risk for developing chronic postoperative pain [4, 5].

Experimentally, the descending inhibitory pathways can be assessed using two noxious stimuli [6]. Plenty of paradigms assessing the conditioned pain modulation (CPM) have been described, using thermal, mechanical, electrical or pressure stimuli as test stimulus (TS) and/ or conditioning stimulus (CS) in different combinations. The so-called CPM-effect is typically calculated as the difference between pain ratings of the TS before and the TS during *or* - to analyse its persistence - after application of the CS [7, 8]. Hitherto, there is no consensus whether a certain CPM protocol is preferable over the others [9].

So far, only few studies have analysed the test-retest-reliability of different CPM paradigms for periods between 15 min and 10 months with sample sizes between 12 and 190 subjects, most of them in healthy subjects, using different TS (heat pain, electrical stimulation, pressure or ischemia) and CS (hot or cold-water baths, occlusion cuff) [10–18]. However, one study focused on intra-individual variances of the CPM-effect elicited by different CS in 12 healthy men [17], while another focused on the influence of ongoing pain on the CPM-effect [18], both of them not examining genuine test-retest-reliability. Three further studies analysed additionally gender-specific test-retest-reliability [13, 16, 18]. To sum up, the results regarding the ICC vary widely between the studies and seem to depend on the used CS and TS and the time interval [7, 16, 17]. For most paradigms and parameters ICC analysis revealed good to excellent test-retest reliability in healthy subjects with some exceptions. Especially for the CPM-effect elicited by cuff occlusion as CS and pressure pain as TS, the ICC revealed poor reliability (ICC −0.4) over a period of 3 days [10], whereas retest with the same CPM paradigm within less than 60 min showed good to excellent ICC [10, 15]. Two studies examining the CPM-effect using electrical stimulation as TS found good test-retest reliability over 1–4 weeks based on both the nociceptive flexor reflex (NFR) response and subjective pain ratings [11, 12], but it was poor when calculated based on the electrical pain detection threshold [12], though the CPM effect based on subjective pain ratings was more reliable than based on the NFR during innocuous stimulation as control condition [11]. The authors concluded that the subjective pain ratings and objective electrophysiological measures reflect different components of the CPM [11, 12]. In contrast, examining the CPM effect in patients with chronic pain over an interval of about 1 week with painful cold stimulus as CS and pressure pain as TS, the test-retest reliability seems to be poor in males, whereas a subanalysis in female patients showed better test-retest reliability according to the ICC [13, 14, 18]. Therefore, extrapolation of reliability measures from one CPM paradigm to another and between different study populations, i.e. healthy subjects vs. patients with chronic pain, seems to be inappropriate. Only two of the above studies reported clinically relevant reliability measures like standard error of measurement and standard real difference [12, 18], though recently a review on studies addressing the test-retest reliability of sensory testing demanded for more detailed statistical evaluations of test-retest data, including also assessment of agreement of the datasets, and more transparent data presentation [19].

To prove the value of a testing paradigm for clinical applicability, an analysis of the test-retest reliability in healthy subjects is an essential prerequisite, as the confounding factors in healthy subjects are less pronounced than in patients with an underlying disease. To our knowledge, a detailed test-retest-reliability analysis in healthy subjects for the commonly used method with tonic heat as TS and tonic cold as CS [7, 20] is still lacking, although this protocol seems to provide clinically relevant information and is easily applicable, e.g. without recording EMG activity. This paradigm was recently applied in patients with painful diabetic neuropathy and was able to identify patients with insufficient endogenous analgesia who were responders to duloxetine, which is supposed to enhance the function of the descending inhibitory pathways by reuptake inhibition of serotonin and noradrenaline [21]. To evaluate the methodological stability of this CPM paradigm for the clinical practice, we analysed its short-term test-retest-reliability (24–72 h) in healthy subjects (primary objective). We analysed the difference between the pain intensity of the TS before and (i) *during* the simultaneous application of the CS (“early CPM-effect”) as well as (ii) *shortly after* the application of the CS (“late CPM-effect”).

Somatosensory function can also be examined by quantitative sensory testing (QST). The QST-protocol of the German Research Network on Neuropathic Pain (DFNS) is reliable and well validated [22–24]. It contains, among others, the determination of thermal and mechanical pain thresholds as well as suprathreshold testing with mechanical pinprick stimuli for measurement of the mechanical pain sensitivity (MPS) and the wind-up ratio (WUR) [22]. Increased pain sensitivity to pinpricks and enhanced wind-up represent two mechanisms of central sensitization, implying a preponderance of the facilitatory pathways [25, 26]. Hence, these QST parameters might also indirectly reflect the state of the descending inhibitory pathways. Therefore, we also analysed their correlation to the magnitude of the CPM-effect (secondary objective).

## Methods

### Subjects

The study protocol was in accordance with the latest version of the Declaration of Helsinki and approved by the local ethics committee of the Faculty of Medicine, Ruhr-University Bochum, Germany (Reg. Nr. 4321–12, NCT01618604).

Volunteers were recruited from June 2012 to December 2012 among students, their relatives or employees of the University Hospital Bergmannsheil in Bochum, Germany. Before starting the assessment, the study was described in its entirety to the subjects, who all gave written informed consent. All subjects received a reimbursement of 70 € for completion of the study. Inclusion criteria were age above 18 years, right-handedness (assessed by the Edinburgh Handedness Inventory [27]), absence of chronic pain, no drug intake, especially no use of analgesic drugs in the last 14 days. Each subject was asked to answer a screening tool for healthy subjects, which has previously been established within the IMI-Europain consortium (www.imi.europa.eu). It comprises the following exclusion criteria: age under 18 years, missing informed consent, insufficient German language skills, current or recent pain, recent intake of analgesic or other drugs (except contraceptives), consume of alcohol or energy drinks, history of neurological, dermatological, chronic internal or psychiatric diseases, abnormal neurological examination, recent sleep restriction or unusual physical exercises, and pregnancy. Relevant depression and anxiety symptoms were additionally excluded by applying the Hospital Anxiety and Depression Scale (HADS) prior to study begin on the first session [28, 29]. Other study-specific exclusion criteria were abnormal results in the baseline quantitative sensory testing (QST) with values outside the 95 % confidence interval for healthy subjects according to the DFNS reference database [24] in order to exclude subjects with incidental or subclinical neuropathy.

One female was excluded because of an abnormal side-to-side difference for thermal detection thresholds, indicating a unilateral neuropathy. None of the subjects was excluded due to one of the above-mentioned criteria concerning the pain ratings during the CPM procedure. Thus, a final sample size of twenty-five subjects (mean age: 31.6 ± 14.3 (21…69) years; 12 females, *n* = 3 > 40 years; 13 males, *n* = 1 > 40 years) was used for further statistical analyses.

### Study design

Subjects attended two sessions 24 to 72 h apart. Both sessions were conducted in exactly the same way and were performed in the afternoon (between 4 pm and 7 pm) by the same female examiner (J.G.) in the certified QST laboratory in the University Hospital Bergmannsheil GmbH in Bochum, Germany. On day 1, each subject answered the German Version of the Pain Sensitivity Questionnaire (PSQ) [30] to assess self-reported pain sensitivity in relation to daily life situations for correlation with the results of the experimental setting (secondary outcome).

**Fig. 1:**
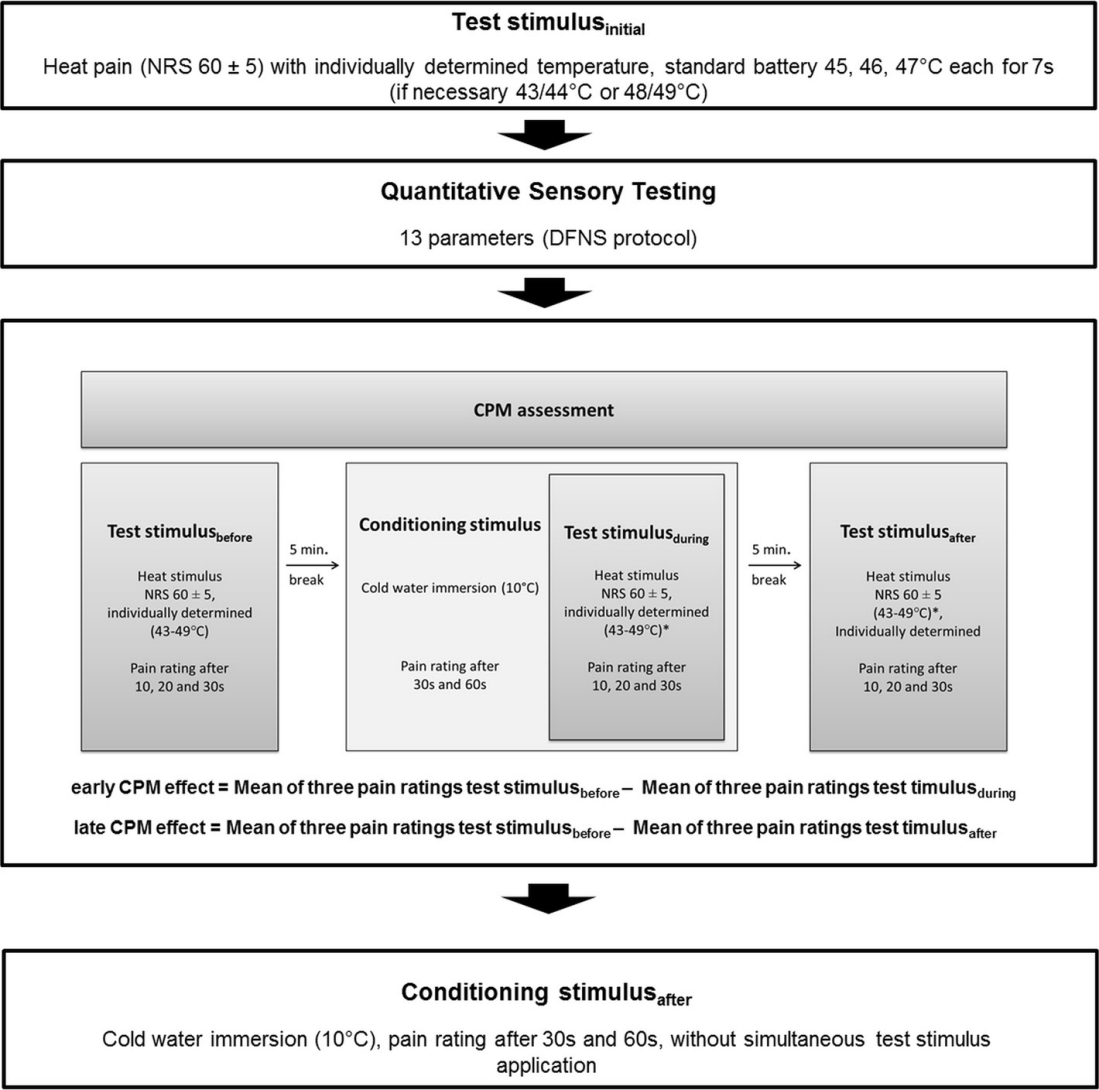
Study design

The test procedure is illustrated in Fig. 1. It strictly adhered to a standardized script with standardized instructions, which were read out to the subjects. The assessment began with the determination of the individual test stimulus temperature (TS_initial_), followed by QST according to the protocol of the German Research Network on Neuropathic Pain (DFNS) on the right and left dorsum of the hand, lasting about 60 min. After that, we applied the established CPM paradigm as previously described by Granot et al. [7] with the initially defined test stimulus (TS) and a fixed conditioning stimulus (CS) (details see below). Adverse events were protocolled during the testing procedure until 30 min after the end of the testing procedures.

The protocols for QST and CPM assessment are adapted from Rolke et al. [22] and Granot et al. [7], respectively. DFNS, German Research Network on Neuropathic Pain; NRS, Numerical Rating Scale; QST, quantitative sensory testing; CPM, conditioned pain modulation.

### Conditioned pain modulation assessment

#### Test stimulus calibration

The TS_initial_ was redefined every day for each subject as the heat stimulus temperature rated with 60 ± 5 on the NRS (0–100) [31].

Heat stimuli were set with a thermal sensory testing device (TSA 2001-II, MEDOC, Israel, CoVAS software, version 3.20) using a thermode with a contact area of 30x30mm and a stimulus ramp of 4 °C/s. First, subjects rated the pain intensity on the NRS (0–100) during a standard heat battery consisting of three stimuli (45, 46 and 47 °C) with a duration of 7 s and an inter-stimulus interval of 35 s to determine the individual TS temperature. The heat stimuli were applied on the right volar forearm and the thermode was moved 30 mm proximally after the first, and 30 mm distally from the middle of the forearm after the second stimulus to prevent thermal sensitization [32]. In case the standard temperatures (45-47 °C) were rated as too painful or not painful enough, two further stimuli of lower or higher temperature, respectively, were used (43 and 44 °C or 48 and 49 °C).

#### Conditioning stimulus

The CS was delivered by immersion of the left hand up to the wrist in a cold-water bath kept at 10 °C for 60s. The temperature was measured by a calibrated quicksilver thermometer (0-100 °C) with an accuracy of ±1 °C. The CS was not individually calibrated, in accordance with the established protocol by Granot et al. [7] and other studies confirming sufficient induction of a CPM-effect with this type and intensity of the CS [10, 33].

#### CPM procedure

The individually determined TS temperature, corresponding to a pain intensity of 60 ± 5 on the NRS (0–100) was applied for 30 s three times during the CPM assessment as TS_before_, TS_during_ and TS_after_ according to the protocol by Granot et al. [7]. Each time, the subjects were asked to rate the heat pain intensity after 10, 20, and 30 s on the NRS (0–100) (see Fig. 1). Five minutes after the first TS (TS_before_), subjects were asked to put their hand into the cold water with spread fingers and without touching the bottom or the walls of the container. Subjects rated the cold pain intensity after 30 and 60 s (NRS, 0–100). After 30 s immersion of the hand in the cold-water bath, the TS was applied simultaneously (TS_during_) for 30 s. Subjects rated the pain intensity again with focus on the TS after 10, 20 and 30 s. Pain ratings for the TS after 30s and the CS after 60s were made separately at the same time, concentrating on the intensity of heat and cold pain, respectively. Finally, 5 min after termination of the CS, the TS was applied again for 30 s (TS_after_) and its intensity was rated after 10, 20 and 30 s. 30 min after the TS_after_, the CS was applied again solitarily for 60 s and its intensity was rated after 30 and 60 s (NRS, 0–100) to analyse distraction effects during simultaneous application of TS and CS.

#### Calculation of the early CPM-effect

The extent of the endogeneous analgesia was calculated as the difference between the mean of the three pain ratings for the TS_before_ (after 10, 20 and 30s) and the mean of the three pain ratings for the TS_during_ (after 10, 20 and 30s), in accordance to previous studies [4, 7] and was defined as early CPM-effect [4, 7].

$$ Early\ CPM- effect = Mean\  of\  three\  pain\  ratings\ T{S}_{before}\hbox{--}\ Mean\  of\  three\  pain\  ratings\ T{S}_{during}. $$

#### Calculation of the late CPM-effect

In order to evaluate the 5-min-persistence of the CPM-effect (late CPM-effect), we furthermore calculated the difference between the mean of the three pain ratings for the TS_before_ and the mean of the three pain ratings for the TS_after._

$$ Late\ CPM- effect = Mean\  of\  three\  pain\  ratings\ T{S}_{before}\hbox{--}\ Mean\  of\  three\  pain\  ratings\ T{S}_{after}. $$

### Quantitative sensory testing

In accordance to the protocol of the German Research Network on Neuropathic Pain (DFNS), the QST assessment included seven tests measuring 13 parameters [22] and was performed on the left and right dorsum of the hand. Before starting the QST assessment, all subjects became familiarized with the stimuli in an area other than the area to be tested. This standardized test battery consisted of the cold detection threshold (CDT), warm detection threshold (WDT), thermal sensory limen (TSL), paradoxical heat sensations (PHS), cold pain threshold (CPT), heat pain threshold (HPT), mechanical detection threshold (MDT), mechanical pain threshold (MPT), mechanical pain sensitivity (MPS), dynamic mechanical allodynia (DMA), wind-up ratio (WUR), vibration detection threshold (VDT) and pressure pain threshold (PPT). For the warm and cold detection the same TSA 2001-II (MEDOC, Israel, Win TSA software, version 5.29) thermal sensory testing device was used as for the CPM assessment (32 °C baseline temperature, stimulus ramp of 1 °C/s, cut-off values 0 °C - 50 °C). The mechanical detection threshold (MDT) was measured using modified von Frey filaments (Optihair2-Set, Marstock Nervtest, Schriesheim, Germany) between 0,25 and 512mN. Modified Pin Pricks (MRC Systems, Heidelberg, Germany) from 8 to 512mN were used for the MPT, WUR and MPS. Light tactile stimuli (cotton wool, brush and Q-tip) were used to assess DMA. The VDT was determined with a Rydel-Seiffer tuning fork (64 Hz, 8/8 scale) that was placed over the ulnar styloid process. The PPT was measured by a pressure algometer placed over the thenar muscle (FDN200, Wagner Instruments, Greenwich, USA, probe area: 1 cm^2^, stimulus ramp 0.5 kg/s, 2-20 kg/cm^2^).

### Statistical analysis

All analyses were conducted using SPSS, Version 20 (SPSS, Chicago, IL, USA). Normal distribution of variables was assured using the Kolmogorov-Smirnov test. Within group differences (e.g., pain ratings day 1 vs. day 2) were analysed using paired t-tests. For analysis of the relative test-retest reliability between the two CPM-assessments, i.e., the degree to which the subjects’ measurements or scores maintained their position relative to others, we used indices of agreement, the Pearson’s product–moment correlation, followed by calculation of the Intraclass Correlation Coefficient (ICC). To control for bias between measurement on both days, ICC analyses were conducted using a two-way mixed effects model with terms of absolute agreement. The standard error of measurement and its 95 % confidence interval, the smallest real difference (SRD) were calculated as absolute measure for the reliability of both the early and late CPM effect (standard error of measurement = intra-individual standard deviation * √(1-ICC), SRD = 1.96 * standard error of measurement * √2$\Big)$ [34]. Both parameters were calculated to reflect the sensitivity of change of the early and late CPM effect. Measuring the magnitude of the CPM effects, it is not only important to calculate the ICC, but also to be able to evaluate changes due to an intervention e.g. a pharmacological treatment. The standard error of measurement indicates the expected error between two measurements conducted under the same circumstances in the same subject over a defined period of time, and should not be confused with the standard error of mean (also abbreviated as SEM). The lower the standard error of measurement, the better the test-retest-reliability [34, 35]. The SRD, as the 95 % confidence interval of the SEM, indicates the change in value that cannot be interpreted as random scatter between two measurements in an individual, but has to be assigned to change of circumstances, i.e. a treatment or intervention effect. Bland-Altman-plots were conducted as a graphical interpretation [36], displaying the relationship between the mean CPM-effect on day 1 and day 2 on the x-axis, and the difference between CPM effects of day 2 and day 1 for each subject on the y-axis, separately for both early and late CPM-effect. An important part of an analysis of reliability is the assessment not only of mean differences, but also of the variance of these differences, as reliability is more important for single subjects than for groups of subjects [37]. In Bland-Altman-plots, both individual effects and effects estimated on the basis of the study group can be seen: the mean difference (i.e. bias) between day 2 - day 1 is marked as a bold line and the estimated 95 % limits of agreement (LoA) as thin lines, and their 95 % confidence intervals as dashed lines. The 95 % LoA represent the range of limits in which 95 % of the data are expected to lie, based on the study population [37], their confidence intervals indicate the level of uncertainty due to the variance in the dataset and the limited number of subjects in the study population. The lower the limits, the closer the values between both measurements and the better the reliability.

As recommended by Biurrun Manresa et al. [12], the clinical relevance of reliability results can become evident by calculating the estimated samples sizes. Therefore we calculated sample sizes for the early and late CPM effect considering crossover (i.e. intragroup reliability analysis, sample size = number of subjects each receiving different assessments) and parallel (i.e. intergroup reliability analysis, sample size = number of subjects for each group) study designs, following the guidelines described by Julious et al. [38]. The calculation is based on the question how many subjects are needed to treat (e.g. drugs, intervention) to elevate a former “non responder” (mean CPM effect = 0) to a “normal responder” (in the case of the present study population: mean early CPM effect = 18 NRS points, and mean late CPM effect = 4 NRS points, see results). For this purpose, we built four subgroups analysing 25, 50, 75 and finally 100 % success of treatment.

Questionnaire data from the PSQ was correlated with the early and late CPM-effect using the Pearson correlation to compare self-reported information on pain sensitivity in daily-life situations and the function of the descending pathways during the experimental setting. Additionally, the HADS scores were correlated with the early and late CPM-effect using the Pearson correlation to assess any association with the magnitude of depression or anxiety scores (within the normal range).

For all analyses comprising QST data, data of the right hand was used. QST raw data was logarithmically transformed, except for PHS, CPT, HPT and PPT, as previously described [24]. All parameters (except PHS and DMA) were z-transformed to compare the data independently from age, gender and region, based on the existing DFNS reference data base [24] using the QST data analysis program eQuiSTA (Casquar GmbH, Bochum, Germany). Test-retest reliability analyses of the QST raw data were performed using the ICC.

Pearson’s product moment correlation was used to assess the association between the QST raw values and the early and the late CPM-effect, respectively. Furthermore, we conducted a median-split analysis, separating subgroups according to the magnitude of the early and late CPM-effect (early CPM-effect: < 15 and ≥ 15; late CPM: effect < 3 and ≥ 3, respectively) and comparing raw data of each QST-parameter (after log-transformation) between these subgroups using the Mann–Whitney-U test in order to detect discrete differences between both subgroups, which do not appear in the correlation analysis considering the whole group.

A *p*-value of 0.05 was considered to be statistically significant, and Bonferroni corrections for multiple comparisons were applied for each test group.

## Results

### Subjects’ characteristics and baseline measures

Measurements were repeated within 40 ± 19.9 h. In all subjects a TS_initial_ with a pain rating of 60 ± 5 on the NRS (0–100) could be determined at both days with a temperature between 45 and 49 °C. 6 subjects had a difference of 1 °C in their TS_initial_ between both days. All subjects could tolerate the 10 °C cold-water bath for 60s. Mild erythema was detected in all subjects in the application area of the thermode, disappearing within 30 min after testing. Two subjects complained about pain up to the shoulder and one about nausea during the cold-water immersion.

### CPM magnitude on day 1 and day 2

**Fig. 2:**
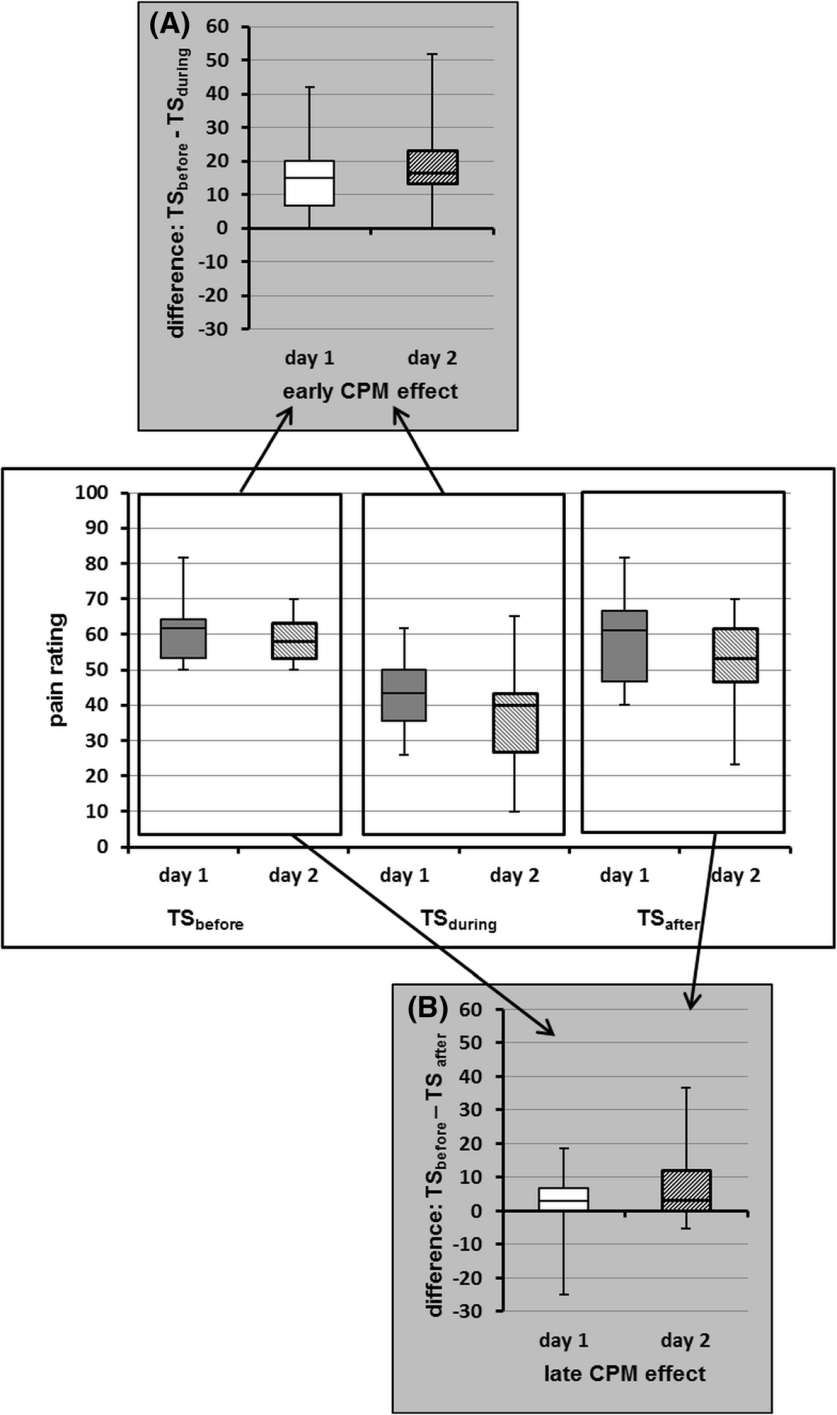
Graphic illustration of the CPM effect’s calculation. **a** Whisker plots of the mean of three pain ratings for TS_before_ and TS_during_, resulting in the early CPM-effect. **b** Whisker plots of the mean of three pain ratings for TS_before_ and TS_after_, resulting in the late CPM-effect. The bottom and the top of the boxes represent the first and third quartiles, the band inside is the median. The ends of the whiskers illustrate the maximum and minimum. TS were applied before, during and after the conditioning stimulus. CPM, conditioned pain modulation; NRS, numeric rating scale; TS, test stimulus

On each day, all but one subject (96 %) had an early CPM-effect >0, indicating that the endogenous pain inhibition during simultaneous cold-water immersion was induced in nearly all subjects. The subject without positive early CPM-effect on both days was not the same. Regarding the whole study group on both days, the pain rating for the TS before application of the CS (TS_before_) significantly decreased during the simultaneous CS application (TS_during_, *p* < 0.01), indicating also a statistically relevant early CPM-effect (see Fig. 2a). The size of the early CPM-effect was 28 on day 1 and 33 % on day 2.

In contrast, a late CPM-effect > 0 was seen only in 14 subjects on day 1 (56 %) and 16 subjects on day 2 (64 %), indicating that endogenous pain inhibition lasted longer than 5 min after termination of the CS only in part of the subjects. 9 subjects (36 %) showed a late CPM-effect on both days, 4 subjects (16 %) presented no late CPM-effect on both days. The group analysis revealed a significant decrease of the pain ratings after CS application (TS_before_ vs. TS_after_) only on day 2 (*p* < 0.01, day 1: *p* = 0.376; see Fig. 2b). The size of the late CPM-effect was 3 on day 1 and 15 % on day 2.

Significant correlations between the magnitude of the pain ratings for the cold water immersion (CS) and the resulting CPM effect could not be observed.

### Test-retest-reliability of conditioned pain modulation

After Bonferroni correction for multiple testing there were no significant differences between both days regarding the reported pain intensities (NRS, 0–100) for TS_initial_, TS_before_, TS_during_, CS_30s_ and CS_60s_, the early CPM-effect or the late CPM-effect (Table 2). Only the pain ratings for TS_after_ were significantly lower on day 2.

Each of the individual parameters of the CPM protocol demonstrated a significant close to moderate correlation between both days based on the ICC (Table 1). Concerning the composite parameters, the correlation of the early CPM-effect between both days was good; however, there was no significant correlation of the late CPM-effect between both days.

**Table 1:** Parameters of the Conditioned Pain Modulation (CPM) assessment

						t-test (p)^a^	Intraclass correlation coefficient^b^
	day 1			day 2		day 1 vs. day 2	
TS temperature rated with NRS 60 (°C)	total	47.5 ± 1.1	(45…49)	47.5 ± 1.0	(46…49)	0.999	0.881 (*p* < 0.001)^**^
Pain rating of TS_initial_	total	64.6 ± 6.8	(55…80)	64.6 ± 8.4	(45…80)	0.974	0.679 (*p* < 0.001)^**^
Mean of three pain rating of TS_before_	total	60.5 ± 7.9	(50…82)	58.4 ± 5.8	(50…70)	0.376	0.411 (*p* < 0.05)
Pain rating of CS_30s_	total	57.7 ± 17.9	(20…90)	56.7 ± 19.3	(20…88)	0.698	0.765 (*p* < 0.001)^**^
Mean of three pain rating of TS_during_	total	43.7 ± 10.0	(26…62)	38.9 ± 14.3	(10…65)	0.018	0.702 (*p* < 0.001)^**^
Pain rating of CS_60s_	total	66.3 ± 20.1	(30…90)	67.5 ± 21.7	(30…95)	0.515	0.906 (*p* < 0.001)^**^
Mean of three pain rating of TS_after_	total	58.8 ± 11.7	(40…82)	50.8 ± 11.7	(23…70)	0.003*	0.480 (*p* < 0.01)
Pain rating of CS_30s after_	total	57.9 ± 18.5	(15…85)	55.6 ± 19.3	(20…95)	0.282	0.843 (*p* < 0.001)^**^
Pain rating of CS_60s after_	total	70.8 ± 19.2	(18…90)	69.5 ± 20.3	(20…95)	0.355	0.946 (*p* < 0.001)^**^
Early CPM effect	total	16.7 ± 11.7	(−5…44)	19.5 ± 11.9	(−3…52)	0.187	0.618 (*p* < 0.001)^**^
Late CPM effect	total	1.7 ± 9.2	(−25…18)	7.6 ± 11.5	(−5…37)	0.035	0.178 (*p* = 0.192)

All values are presented as mean ± SD and range. All data represent pain ratings on the numeric rating scale (NRS, 0–100), unless otherwise indicated. *CS* conditioning stimulus, *TS* test stimulus; *CPM* conditioned pain modulation. The early CPM effect is calculated as the difference between mean pain ratings of TS_before_ and TS_during_. The late CPM effect is calculated as the difference between the mean pain ratings of TS_before_ and TS_after_

^a^paired t-test CPM-effect day 1 vs. day 2

^b^Intraclass Correlation Coefficient (ICC) between CPM parameters day 1 and day 2

*p < 0.005 (after Bonferroni correction)

^**^ *p* < 0.005 (after Bonferroni correction)

For the early CPM-effect the standard error of measurement (SEM) was 7.3 on the 0–100 NRS scale, representing 40 % of the mean CPM-effect (Table 2). For the late CPM-effect, the SEM was 211 % as big as the mean (Table 2). The smallest real difference (SRD) indicated that a change larger than 20.2 NRS points for the early CPM effect, and 27.0 NRS points for the late CPM effect in an individual case has to be assigned to a real change and not random scatter between measurements (Table 2).

**Table 2:** Test-retest-reliability analyses for the mean early and late CPM effect (day 1 vs. day 2), *n* = 25

	Mean	SD	Bland-Altman analysis bias (lower LoA-upper LoA)	ICC (95 % CI)	SEM	SRD
early CPM effect	18.1	11.8	2.8 (−17.4… 23.0)	0.618 (0.302-0.811)	7.3	20.2
late CPM effect	4.6	10.7	6.0 (−20.2… 32.1)	0.178 (−0.226-0.530)	9.7	27.0

*CPM* Conditioned Pain Modulation, mean values for the CPM effect day 1 and day 2 represent pain ratings on the numeric rating scale (NRS, 0–100). SD; standard deviation, *ICC* Intraclass Correlation coefficient, *CI* confidence interval, *LoA* limits of agreement, *SEM* standard error of measurement, *SRD* smallest real difference. The standard error of measurements has the same units as the CPM measurements (NRS, 0–100) and is calculated as SEM = SD * √1-ICC. The SRD represents the 95 % confidence interval of the SEM, i.e., SRD = 1.96 * SEM * √2. For this table we assembled the mean values for the early and late CPM effect on both test days

Given the fact, that a CPM “non-responder” might have a CPM effect of 0, and a “normal responder” might have a mean CPM effect with 18 NRS points for the early CPM effect, and 4 NRS points for the late CPM effect (Table 2), we calculated hypothetical sample sizes for crossover and parallel designs in terms of successful treatment (Table 3). In case of 50 % treatment success after an intervention, 18 subjects are needed to confirm this hypothesis in the crossover design, and 68 subjects in the parallel design for the early CPM effect. For the late CPM effect the number of subjects needed to confirm such a hypothesis is beyond the clinical relevance.

**Table 3:** Hypothetical sample size calculations for crossover and parallel designs in terms of successful treatment

Patients return to normal CPM effect (rate of treatment success)	Early CPM effect		Late CPM effect	
	Crossover	Parallel	Crossover	Parallel
100 %	6	18	45	176
75 %	10	34	122	484
50 %	18	68	272	542
25 %	84	330	1084	4332

*CPM* Conditioned Pain Modulation. Calculations are based on the question how many subjects are needed to treat to elevate “non responders” to “normal responders” in case of 25/ 50/ 75/ 100 % treatment success. All values are given as the number of subjects for each group

**Fig. 3:**
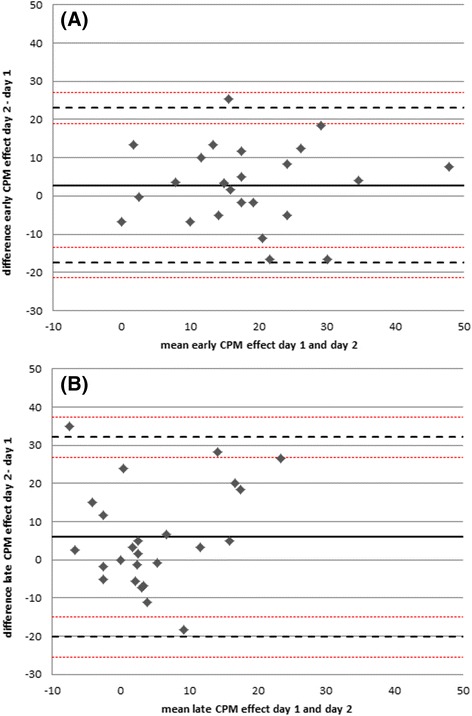
Bland-Altman plot for the CPM-effect on day 1 and the difference between the CPM-effect on day 2 and day 1. **a** early CPM-effect (*r* =0.019, *p* = 0.928), **b** late CPM-effect (*r* = 0.215, *p* = 0.302). The bold line is the mean difference of the CPM-effect of both days, the dashed lines represent the 95 % limits of agreement. CPM, Conditioned Pain Modulation; NRS, numeric rating scale

The Bland-Altman Plot for the early CPM effect (Fig. 3a) showed a range for the 95 limits of agreement (LoA) between −17.4 (95 CI: −21.5…-13.4) NRS points (0–100) and 23.0 (95 % CI: 19.0…27.1) NRS points. The late CPM effect (Fig. 3b) showed a range for the 95 LoA between −20.2 (95 CI: −25.4… -15.0) NRS points and 32.1 (95 % CI: 26.9… 37.3) NRS points (Table 2). Regarding the absolute range, the limits for the late CPM effect were approximately 30 % wider than for the early CPM effect. Figure 3a shows all values evenly distributed around the mean difference (bold line = bias), 11/25 subjects are below the mean difference, 14/25 subjects above, indicating no systematic deviation between the measurements, that could be assigned, for example, to learning effects (correlation *r* = 0.019, *p* = 0.928). The mean early CPM effect between both days ranges between 0–50 NRS points, the mean difference between −17 and + 18 NRS points. The range for the mean late CPM effect between both days is between −8 and 25 NRS points, the mean difference between −19 and +33 NRS points.

The early and late CPM-effect did not correlate significantly neither on day 1 (*r* = 0.330, *p* = 0.107) nor on day 2 (*r* = 0.375, *p* = 0.065).

### Quantitative sensory testing

#### Test-retest reliability of quantitative sensory testing

After z-transformation, all subjects showed QST parameters within the normal range between −1.96 and 1.96, as expected. One male subject (63 years) reported 1 (of 3) paradoxical heat sensations on day 1. None of the subjects had dynamic mechanical allodynia.

In the whole study group, all QST parameters correlated significantly between day 1 and day 2 (ICC = 0.450…0.916) except for the cold detection threshold (CDT, ICC = 0.265).

#### Correlation of quantitative sensory testing and CPM-effects

There were no significant correlations between the magnitude of the early and late CPM-effect and all QST parameters, which were present at both days. After Bonferroni correction, the correlation between the heat pain threshold (HPT) and the late CPM-effect on day 2 was not significant (*r* = − 0.401, *p* = 0.047, Table 4). Likewise, median split analyses regarding the early and late CPM-effect revealed no significant differences between all QST parameters, which occurred on both days, especially regarding the thermal (CPT: *p* = 0.609…0.936, HPT: *p* = 0.344…0.936) and mechanical pain thresholds (MPT: *p* = 0.244…0.979, PPT: *p* = 0.205…0.467) and parameters of the stimulus–response-function (MPS: *p* = 0.046…0.809, WUR, *p* = 0.470…0.979).

**Table 4:** Correlational analyses between CPM effects and QST parameters at day 1 and day 2

	Pearson’s correlation coefficient				
		QST vs. early CPM effect day 1	QST vs. early CPM effect day 2	QST vs. late CPM effect day 1	QST vs. late CPM effect day 2
CDT (log)	total	0.008 (*p* = 0.969)	−0.080 (*p* = 0.704)	−0.143 (*p* = 0.497)	0.311 (*p* = 0.131)
WDT (log)	total	−0.175 (*p* = 0.402)	−0.017 (*p* = 0.935)	−0.125 (*p* = 0.553)	−0.133 (*p* = 0.528)
TSL (log)	total	−0.136 (*p* = 0.517)	−0.096 (*p* = 0.648)	0.108 (*p* = 0.608)	0.088 (*p* = 0.675)
CPT (°C)	total	0.059 (*p* = 0.778)	0.158 (*p* = 0.449)	0.055 (*p* = 0.795)	0.349 (*p* = 0.088)
HPT (°C)	total	−0.103 (*p* = 0.624)	−0.087 (*p* = 0.679)	0.065 (*p* = 0.757)	−0.401 (*p* = 0.047)
MDT (log)	total	−0.103 (*p* = 0.624)	0.042 (*p* = 0.840)	0.057 (*p* = 0.787)	0.122 (*p* = 0.561)
MPT (log)	total	0.210 (*p* = 0.314)	−0.020 (*p* = 0.923)	0.203 (*p* = 0.330)	−0.213 (*p* = 0.308)
MPS (log)	total	0.049 (*p* = 0.815)	−0.202 (*p* = 0.333)	−0.142 (*p* = 0.500)	0.020 (*p* = 0.926)
WUR (log)	total	−0.118 (*p* = 0.574)	−0.200 (*p* = 0.338)	−0.042 (*p* = 0.843)	0.211 (*p* = 0.312)
VDT (n/8)	total	0.317 (*p* = 0.122)	0.336 (*p* = 0.100)	0.217 (*p* = 0.297)	−0.129 (*p* = 0.538)
PPT (log)	total	−0.085 (*p* = 0.687)	−0.011 (*p* = 0.959)	0.184 (*p* = 0.380)	−0.337 (*p* = 0.100)

Correlational analyses between each QST parameter (logarithmically transformed, except for *CPT* HPT, VDT) and the CPM effect’s magnitude were conducted using Pearson’s Correlation Coefficient for measurements at day 1 and day 2. *CDT* Cold Detection Threshold, *CPT* Cold Pain Threshold, *HPT* Heat Pain Threshold, *MDT* Mechanical Detection Threshold, *MPS* Mechanical Pain Sensitivity; *MPT* Mechanical Pain Threshold, *PPT* Pressure Pain Threshold, *QST* Quantitative Sensory Testing, *TSL* Thermal Sensory Limen; *VDT* Vibration Detection Threshold, *WDT* Warm Detection Threshold, *WUR* Wind up Ratio

Level of significance after Bonferroni correction: p<0.001

### Questionnaires

The scores for the HADS were within the range for healthy subjects (subscore for anxiety: 3.0 ± 2.2 (0…5); subscore for depression: 1.4 ± 1.7 (0…5)). The mean PSQ scores (3.4 ± 1.4 (0.6…6.1) were similar to the data for healthy subjects originally reported by the authors (3.6 ± 1.2 [30]).

Regarding the whole study group, no significant correlations were found between PSQ, HADS overall score and subscores for anxiety and depression and the early or late CPM-effect.

## Discussion

To summarize, we induced endogenous pain inhibition with CPM-effect >0 using cold-water immersion as CS in almost all healthy subjects on both days. However, in >1/3 of them it lasted for less than 5 min after the CS termination. The absolute magnitude of the early CPM-effect (during simultaneous CS application) was similar on both days and was consistent with most of the previous studies [20], though in contrast to some others, which reported lower [39, 40] or higher CPM-effects [17, 41, 42]. For the early CPM-effect, the test-retest-reliability within the period of 24-72 h assessed in healthy subjects showed a SEM of about 40 % of the mean and a SRD of 20 (on the NRS 0–100). In contrast, the reliability of the late CPM-effect, assessed 5 min after CS termination, was rather insufficient, represented in a SRD nearly six times bigger than the mean effect, making the detection of a real change between two assessments within a subject nearly impossible and estimated sample sizes for experimental studies rather unrealistic. Neither thermal nor mechanical pain thresholds, nor QST parameters comprising suprathreshold stimuli correlated with either CPM-effect.

### Differences between the CPM paradigms regarding the test-re-test-reliability

So far, nine studies have been published analysing the test-retest reliability of different CPM paradigms, than the one we used [10–18]. Three of them analysed the reliability in chronic pain patients or in an experimental pain model for acute musculoskeletal pain [13, 14, 18]. The remaining six studies analysed the test-retest-reliability in healthy subjects using the nociceptive withdrawal reflex (NWR), electric, pressure or heat pain as TS and cold- or hot-water baths and ischemia as CS. Three studies reported similar ICC as in our study ranging between 0.54 and 0.69 [10, 11, 15]. However, two studies reported insufficient reliability based on ICC: Biurrun et al. used NWR thresholds, electrical pain thresholds and suprathreshold electrical pain as TS and cold-water bath as CS with ICC of 0.09 to 0.44 within 1–3 weeks for suprathreshold electrical pain [12]. Another study in healthy females used heat both as TS and CS and achieved ICC = 0.39 for retests over a period of 7–10 months [16]. In contrast, our study concentrated on the short-term test-retest-reliability, which might explain the better ICC, pointing to a more stable CPM-effect over shorter periods of time. Choosing an appropriate time period for reassessment is an important aspect. While re-assessment over very short time periods of 60 min or less [Cathcard2009; Lewis2012] might be more reliable, very short time periods might be insufficient to expect a real treatment effect when examining e.g. changes in the CPM-effect due to an intervention. On the other hand longer time periods between reassessments implicate changed external conditions which might influence the results, especially when examining patients, also in relatively short term of about 1 week [13, 14] which is pronounced in male patients [13]. There is also some evidence, that different CPM paradigms engage different spinal or supraspinal inhibitory mechanisms [43–45]; e.g., the magnitude and stability of CPM-effects with the use of subjectively reported pain intensities was shown to be stronger than objective measures like the NWR [44, 45].

Furthermore, most studies reported only ICC values as measure for test-retest-reliability [10, 11, 13–16]. One study showed higher ICC values for the CPM-effect based on subjective pain ratings compared to electrophysiological responses during the NWR (ICC 0.44 vs 0.26, [12]), while another reported similar ICC values over 28 days for both TS (ICC 0.54 vs 0.61, [11]). It has been previously discussed that cognitive influences may represent a stable confounder between test and retest session, thus explaining the slightly better reliability in our study in comparison to a study examining the CPM elicited by the same CS based on electrophysiological measures [12]. Only a few studies conveyed ICC analysis also of the single parameters during CPM assessment, i.e. of CS as well as TS before, during and after CS application). In terms of the ICC, the pain rating of the CS in our study indicated excellent reliability with higher values (ICC 0.77-0.95) than previously reported when using the same CS in patients with chronic pain (ICC 0.61-0.67) [13] and similar ICC as when heat was used as CS (ICC 0.79) [16]. Though tonic heat has been previously suggested to be more constant and less confounded by changes in cardiovascular activity in healthy subjects compared with cold water stimuli [11, 46], our results suggest that both conditioning stimuli are at least comparable regarding their retest-reliability. The current evidence suggests that both tonic cold and heat stimuli as CS seem to be superior to cuff occlusion as CS, as the latter was able to induce a reliable CPM effect only up to 60 min, but not over a period of 3 days, [10, 15].

Only two reported SEM and SRD values [12, 18], which allow much more realistic estimation of the clinical relevance in terms of evaluating the outcome after intervention based on the CPM-effect. While Biurrun et al. [12] studied a completely different CPM-paradigm with electrical stimulation as TS, Valencia et al. assessed similar protocol as ours with repeatedly applied heat pulses as TS and cold pain as CS and reported a minimal detectable change of about 17 in the healthy cohort for re-test measurement within two minutes independently on the fact whether additionally the healthy subjects experienced exercise induced muscle pain or not [18].

For the first time, we report a smallest real difference for the commonly used protocol within a re-test period of 24-72 h, an interval which might be more relevant in term of evaluation a therapeutic interventions based on the CPM-effect. Our results depict that, using the presented CPM-protocol with respect to the early CPM-effect, a smallest real difference more than 20 points on the 0–100 NRS between two measurements in a healthy individual is relevant and can be assigned to change of circumstances, and is not a random scatter. In accordance to our findings, in a subgroup of patients with polyneuropathy, who did not have any pain relief after duloxetin treatment, presented with a more efficient CPM already prior to treatment and the CPM-effect changed on average about 10 on a 101-point NRS after treatment [21]. In contrast, in the subgroup of patients with sufficient pain relief after treatment with duloxetin, the change in CPM-effect was on average 15. This represents a value below the calculated SRD in our study and indicates that in pathological states with impaired CPM even smaller changes than in healthy states, might be clinical relevant. On the other hand, it implicates that further studies examining the test-retest-reliability in patients with chronic pain and CPM impairment without intervention between the measurements are needed to strengthen this hypothesis.

Only one study evaluated the reliability of a CPM paradigm by calculating sample sizes for potential experiments, thus using a clinically more relevant parameter than the pure calculation of the ICC [12]. Under the experimental conditions described here, the sample sizes and consequently the reliability of the early CPM-effect are certainly acceptable and realistic for experimental or clinical use, especially for a crossover design (Table 3). For example, in case of an intervention, which is intended to normalize the CPM-effect in 50 % of the treated patients, who were previously incapable to activate their CPM, one would need a study sample of at least 18 subjects for crossover and 68 subjects for parallel design to detect a significant change after the intervention. Such estimations for calculating hypothetical sample sizes should be demanded also for other studies on re-test-reliability, as they provide a much more realistic measure for the clinical relevance of the data than statistical measures like the ICC.

### Persistence of the CPM effect after termination of the conditioning stimulus

In our study, the late CPM-effect, i.e. endogenous inhibition lasting for at least 5 min after CS termination, was smaller than the early CPM-effect, in line with previous studies [8, 41, 47, 48]. It also varied largely between both days, though statistically not significant after Bonferroni correction, resulting in its insufficient test-retest-reliability and unacceptably high sample sizes in comparison to those for the early CPM-effect to detect the same effect. Even though there were no correlations between the magnitude of the CS rating and the resulting CPM-effect, one might argue that the early CPM-effect is simply the result of distraction by the painful cold-water bath. Though, combined distraction and CPM induced greater pain reduction than either alone [49]. Moreover, different extents of cortical activation in frontal and somatosensory areas were found by distraction and by CPM [50]. Our findings point to an influence of distraction on CPM, but nevertheless support the existence of a “real” CPM-effect, which however seems to have a higher interindividual variability. One study reported no late CPM-effect at all [51], whereas another found greater and longer lasting effects of pain inhibition [52], both assessing the CPM-effect as change in the TS intensity (pain threshold) and not of the pain intensity as in our protocol. These inconsistent findings indicate that the duration of the CPM-effect after CS termination might differ depending on the applied stimuli, but also depending on the chosen read-out, i.e. change in pain intensity of a predefined stimulus or change of pain threshold.

### Correlations between CPM and detection/ pain thresholds as well as suprathreshold stimulation

Another objective of the present study was to assess possible correlations between the CPM-effects and parameters of a standardized QST-protocol. Theoretically, subjects can be positioned in a continuum between pro- and anti-nociception, e.g., a “pro-nociceptive subject” would have a low CPM-effect and show low pain thresholds and high pain ratings for suprathreshold stimuli in his sensory profile [9]. Surprisingly, we found no correlations between QST parameters and the early or late CPM-effect. Previous studies on CPM using pain thresholds as TS showed sufficient CPM-effects resulting in pain threshold increase [2, 53, 54]. However, whilst others analysed pain thresholds as TS, i.e. read-out for the CPM-effect, we analysed for the first time correlations between the sensory profile and the CPM-magnitude. Despite applying the same stimuli, our CPM paradigm uses suprathreshold stimuli, whereas during the QST according to the DFNS-protocol mainly subthreshold stimuli are applied, i.e. until the first perception of pain. These thermal and mechanical pain thresholds did not correlate with the early and late CPM-effect. Suprathreshold stimulation within the DFNS-protocol is only conducted using pinpricks assessing the mechanical pain sensitivity and the wind-up ratio, but they were also not associated with the CPM-effects, probably pointing to different neuronal pathways [21, 22, 43, 55]. Given the high reliability of the QST parameters of the DFNS-protocol [23], replicated in this study, the rather large variability of the CPM magnitude seems not to influence the parameters of the sensory profile.

### Influencing factors on the magnitude of the CPM-effect

Depression and anxiety have been shown to partly influence pain thresholds and suprathreshold testing [55–59] and are linked to neurotransmitters deficiency including serotonin and dopamine, which are also involved in the descending nociceptive inhibitory pathways [60–62]. As expected, in our sample of heathy subjects all HADS scores were within the normal range [29, 63]. Thus, analysing the relationship between these psychiatric symptoms and CPM was not diagnostically conclusive, though there was no correlation with the early or the late CPM-effect. Also the PSQ exploring the perception of potential daily life pain [30] did not correlate with CPM-effects. This was somehow unexpected, as the PSQ score was reported to correlate significantly with experimental pain ratings in healthy subjects having similar PSQ scores as our study population [30]. However, because PSQ-score correlates with QST parameters [30] and QST does not correlate with CPM-effects in the present study our results are not unexpected. For pain patients any associations to both psychometric scales should be further explored.

### Limitations

In our study, we used an established protocol [7], whereupon the TS calibration was conducted with a 7 s tonic heat stimulus, while the TS during the CPM procedure lasted 30 s. Time is a critical parameter with regard to pain intensity, as either habituation or sensitisation can occur during application of long-lasting stimuli. In future studies, the same length of TS application for the calibration and during CPM-testing should be considered. Another point was that the CS was not individually calibrated. Though the used cold-water immersion was reported to be a sufficient CS [7, 17], the high variability of the perceived cold pain intensity might account for the lack of individualized CS calibration. As a psychophysical method, CPM is susceptible to emotional and cognitive factors, such as expectation [64, 65], stress [40] or distraction [40, 49], which might play a role in our study as subjects were not initially familiarized with the CPM procedure. This might explain the higher CPM-effects on day 2, when they were more accustomed to the situation. For future studies, familiarization with the experimental set-up should be obligate. Also experienced and non-experienced subjects should not be included into one study, and information about the familiarization procedures should be reported in detail.

The lack of control task is another limitation of the study, because a pain habituation after repeated application of the TS, accounting partly for the observed pain reduction, cannot be excluded, as thermal heat pain applied by a thermode significantly habituated within the first 6 stimuli [66]. Generally, one main critic against the concept of conditioned pain modulation is the fact that the CPM-effects might be just the result of peripheral habituation or distraction (see above). However, it could be demonstrated that the CPM-experimental paradigm produced significantly more pain reduction than the habituation paradigm and the paradigm involving non-noxious inhibitory control [67]. Granot et al. [7] using the very same CPM protocol as we did, have also found that only immersion of the dominant hand in 12 °C cold and 46.5 °C hot water, but not in the conditions with less painful cold or warm water, elicited a significant CPM-effect comparable to that in our study.

Due to organisational reasons the time interval between both CPM-assessments ranged between 1–3 days. Thus, possible learning effects might have influenced the results of subjects with 24-h-intervals in-between CPM-assessments differently than those with longer breaks. Our aim was not to analyse gender differences, which explains the comparatively small chosen sample size. The sample variability within the group with subjects between 21 and 69 years, might have been quite high, as it is well known that age and sex can affect attentional processes. Though, to minimize such effects, we complemented our analyses for the standard error of measurement to compare not only interindividual, but also intraindividual differences. Furthermore we did not specify the menstrual cycle phase in all female subjects. On this issue, there exist divergent opinions: Rezaii et al. showed that female sex hormones modulate CPM [68], while Wilson et al. demonstrated no variations between CPM during the menstrual cycle [16]. Further reliability studies using the heat-cold-pain method for CPM are needed with a larger sample size to analyse gender and differences regarding the retest-reliability and differences during the menstrual cycle, as appropriate. Additionally, the retest-reliability for longer time-intervals such as weeks or months should be also further examined, which are more relevant for follow-ups in chronic pain patients. For principle reasons, the validity of this method in patients cannot be evaluated in healthy subjects. Accordingly, also the clinical relevance of the early and especially of the late CPM-effect needs further exploration.

## Conclusions

In conclusion, we evaluated the most commonly used method for CPM with heat and cold pain as TS and CS, respectively, demonstrating a sufficient reliability for the early CPM-effect and associated parameters within 48 h, but not for the late CPM-effect. Based on the SRD and SEM as well as considering the above-mentioned limitations, sample size calculations for studies using CPM-effect, evaluated during simultaneous application of tonic heat as TS and tonic pain as CS, as a primary outcome are realistic for experimental or clinical use. Based on our hypothetical sample size calculations crossover design should be preferred rather than paralleled design, due to the high interindividual variability of endogenous analgesia.
